# Supplementary material for: Thai Native Chicken as a Potential Functional Meat Source Rich in Anserine, Anserine/Carnosine, and Antioxidant Substances
Source: Animals (Basel). 2021 Mar 22;11(3):902. doi: 10.3390/ani11030902 (PMC8004088; doi:10.3390/ani11030902)
Supplement: Supplementary file 1 [file animals-11-00902-s001.pdf]

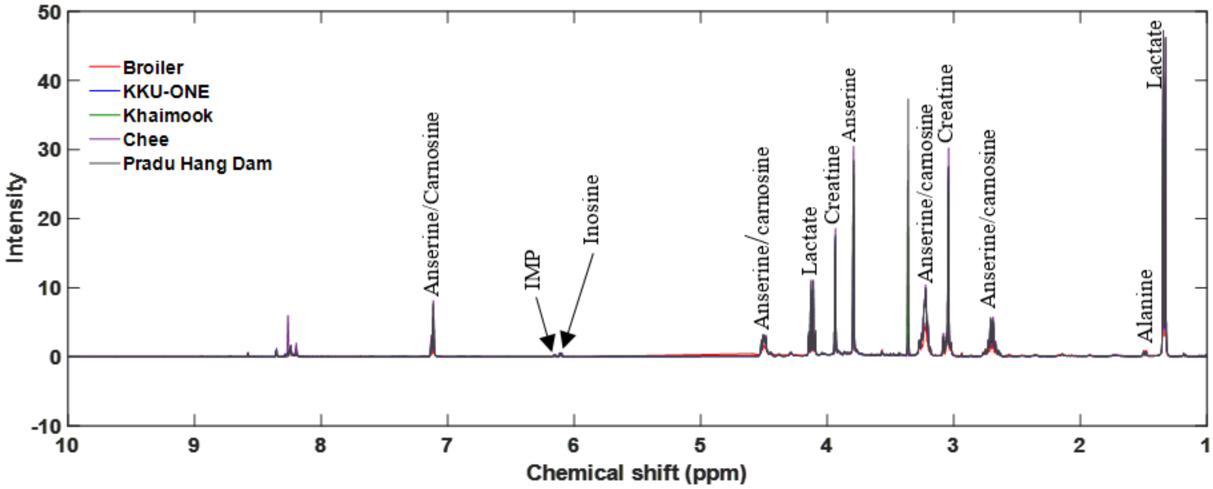

Figure S1. Representative 400-MHz ^{1}H NMR spectra of Thai native chickens (Pradu Hang Dam Mor Kor 55 and Chee KKU 12), Thai synthetic chicken s (Khai Mook Esarn KKU50), Thai native crossbred chickens (KKU-ONE), and commercial broilers (Arbor Acer).
